# Gait and Balance as Predictors or Mediators of Falls in Glaucoma

**DOI:** 10.1093/geroni/igaa057.2784

**Published:** 2020-12-16

**Authors:** Aleksandra Mihailovic, Regina De Luna, Sheila West, David Friedman, Laura Gitlin, Pradeep Ramulu

**Affiliations:** 1 Johns Hopkins University/Wilmer Eye Institute, Baltimore, Maryland, United States; 2 Wilmer Eye Institute/Glaucoma Center of Excellence, Baltimore, Maryland, United States; 3 Johns Hopkins School of Medicine, Baltimore, Maryland, United States; 4 Massachusetts Eye and Ear, Harvard Medical School, Boston, Massachusetts, United States; 5 Drexel University, Philadelphia, Pennsylvania, United States

## Abstract

Balance and gait are modifiable targets for falls prevention and may play an important role in preventing falls in older visually impaired individuals. Balance and gait were objectively evaluated in the 239 Falls in Glaucoma Study participants (average age=70.5, 22% with moderate-severe visual field (VF) damage). Greater sway, more time in double support and greater swing time variability were associated with higher fall rates, while higher gait velocity and faster cadence were associated with lower rates (p<0.05 for all). Neither gait nor balance mediated the relationship between VF damage and fall rates, with VF damage remaining an independent predictor of fall in models including gait and/or balance features (RR =1.36 to 1.48, p-value= <0.001 to 0.005). While balance and gait measures are associated with fall rates, they do not explain why persons with greater VF damage fall more frequently, suggesting the importance of other factors such as hazard perception.

